# Supplementary material for: Transcriptomic Insights into Mechanisms of Early Seed Maturation in the Garden Pea (Pisum sativum L.)
Source: Cells. 2020 Mar 23;9(3):779. doi: 10.3390/cells9030779 (PMC7140803; doi:10.3390/cells9030779)

Zhongwan−6,25(2)

Global Summary

%DE = 0.26

genes with fdr < 0.2 = 6806 (3078 + / 3728 −)

genes with fdr < 0.1 = 6215 (2811 + / 3404 −)

genes with fdr < 0.05 = 5839 (2651 + / 3188 −)

genes with fdr < 0.01 = 5156 (2341 + / 2815 −)

genes in genesets = 0

<FC> = 0

<t−score> = 0

<p−value> = 0

<fdr> = 0.74

Global Genelist

Rank	ID	log(FC)	p−value / fdr	Metagene	Description
1	TRINITY_DN2	−0.06	[illegible]		
2	TRINITY_DN1	0.05	[illegible]		
3	TRINITY_DN4	0.05	[illegible]		
4	TRINITY_DN4	0.04	[illegible]		
5	TRINITY_DN4	−0.15	[illegible]		
6	TRINITY_DN3	−0.03	[illegible]		
7	TRINITY_DN3	−0.05	[illegible]		
8	TRINITY_DN3	−0.04	[illegible]		
9	TRINITY_DN3	−0.04	[illegible]		
10	TRINITY_DN3	0.05	[illegible]		
11	TRINITY_DN3	0.04	[illegible]		
12	TRINITY_DN3	0.05	[illegible]		
13	TRINITY_DN1	−0.1	[illegible]		
14	TRINITY_DN1	0.03	[illegible]		
15	TRINITY_DN1	−0.14	[illegible]		
16	TRINITY_DN1	−0.03	[illegible]		
17	TRINITY_DN1	−0.13	[illegible]		
18	TRINITY_DN1	0.08	[illegible]		
19	TRINITY_DN5	−0.04	[illegible]		
20	TRINITY_DN1	0.11	[illegible]		

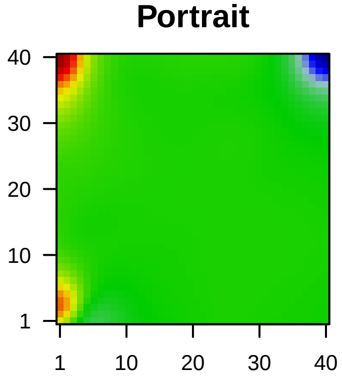

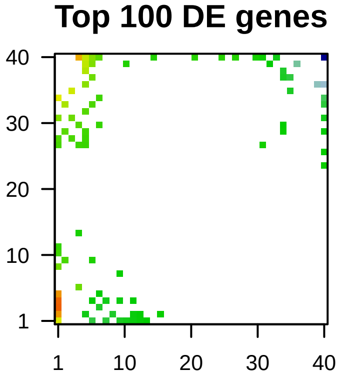

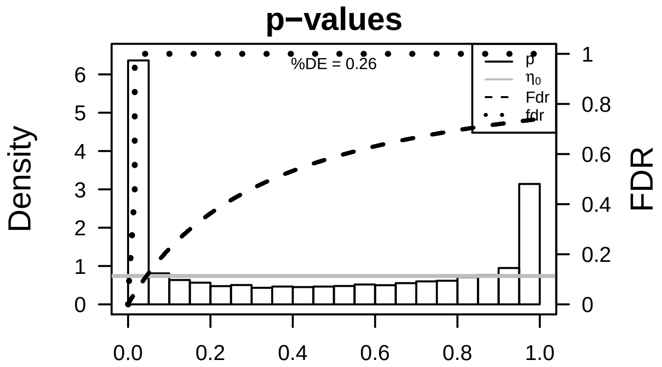

Supplement: Supplementary file 1 [file cells-09-00779-s001.zip › Supplementary materials/FileS2.zip!/FileS1/Summary Sheets - Samples/Zhongwan.6.25.2..pdf]
